# Sensitivity, Noise and Resolution in a BEOL-Modified Foundry-Made ISFET with Miniaturized Reference Electrode for Wearable Point-of-Care Applications

**DOI:** 10.3390/s21051779

**Published:** 2021-03-04

**Authors:** Francesco Bellando, Leandro Julian Mele, Pierpaolo Palestri, Junrui Zhang, Adrian Mihai Ionescu, Luca Selmi

**Affiliations:** 1 Electronic Department, Swiss Federal Institute of Technology Lausanne, 1015 Lausanne, Switzerland; francesco.bellando@epfl.ch (F.B.); adrian.ionescu@epfl.ch (A.M.I.); 2 DPIA Department, University of Udine, 33100 Udine, Italy; mele.leandrojulian@spes.uniud.it; 3 Xsensio SA Batiment A, EPFL Innovation Park, 1015 Lausanne, Switzerland; junrui.zhang@xsensio.com; 4 DIEF, University of Modena and Reggio Emilia, 41125 Modena, Italy; luca.selmi@unimore.it

**Keywords:** ISFETs, electrical characterization, noise, transient behavior, DC behavior

## Abstract

Ion-sensitive field-effect transistors (ISFETs) form a high sensitivity and scalable class of sensors, compatible with advanced complementary metal-oxide semiconductor (CMOS) processes. Despite many previous demonstrations about their merits as low-power integrated sensors, very little is known about their noise characterization when being operated in a liquid gate configuration. The noise characteristics in various regimes of their operation are important to select the most suitable conditions for signal-to-noise ratio (SNR) and power consumption. This work reports systematic DC, transient, and noise characterizations and models of a back-end of line (BEOL)-modified foundry-made ISFET used as pH sensor. The aim is to determine the sensor sensitivity and resolution to pH changes and to calibrate numerical and lumped element models, capable of supporting the interpretation of the experimental findings. The experimental sensitivity is approximately 40 mV/pH with a normalized resolution of 5 mpH per µm^2^, in agreement with the literature state of the art. Differences in the drain current noise spectra between the ISFET and MOSFET configurations of the same device at low currents (weak inversion) suggest that the chemical noise produced by the random binding/unbinding of the H^+^ ions on the sensor surface is likely the dominant noise contribution in this regime. In contrast, at high currents (strong inversion), the two configurations provide similar drain noise levels suggesting that the noise originates in the underlying FET rather than in the sensing region.

## 1. Introduction 

**Figure 1:**
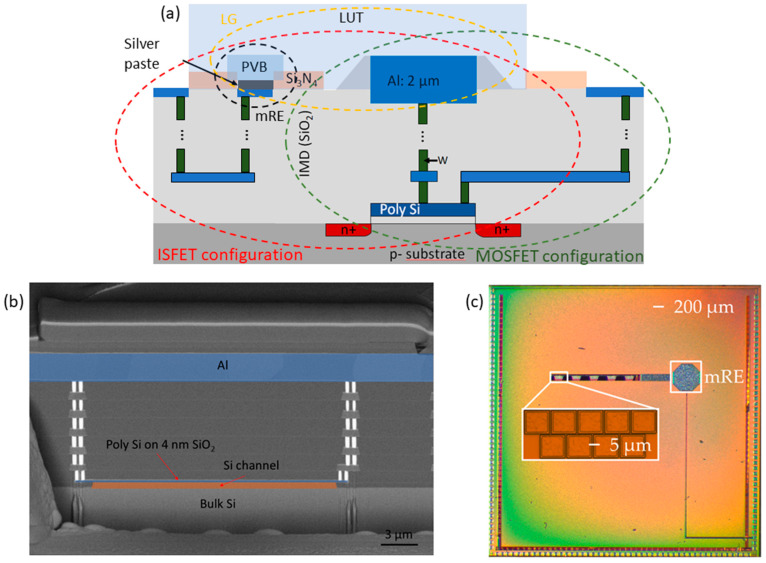
Modified-back-end of line (BEOL) by post-processing a foundry-made complementary metal-oxide semiconductor (CMOS) chip to allow for a liquid under test (LUT) experiment aiming at pH sensing. (**a**) Schematic cross-section of the FET/ISFET, showing how the same device can be characterized in two configurations. (**b**) TEM image of a section of the foundry-made FET, before BEOL modification. This version of the device has a wider Al contact than the one used in our study, but was otherwise identical. (**c**) Optical image of the top view of the sensing chip, showing the base contact for the miniaturized reference electrode (mRE) and the top gates of the five sets of nine ISFETs.

Ion-sensitive field-effect transistors (ISFETs) are a class of potentiometric sensors experiencing a steady growth in interest from the industry and the sensor scientific community, due to their reduced size, ultralow-power consumption, and compatibility with mainstream complementary metal-oxide semiconductor (CMOS) technology, which make them extremely suitable for the exploding field of large scale distributed wearable diagnostics and Point-of-Care analysis [1,2,3,4,5]. Indeed, the schematic structure of an ISFET is almost identical to the one of a conventional MOSFET (Figure 1a), except that the ISFET gate terminal is a liquid gate (LG) composed of a reference electrode (RE) immersed in a liquid under test (LUT) that wets a sensing layer electrically connected to the gate of an underlying transistor, instead of a simple solid-state conductive line. This similarity between the two structures allows us to characterize the very same device in ISFET or FET configuration by simply selecting the metal gate or LG contact: Any difference between the measured parameters can therefore be attributed solely to the liquid gate (see Figure 1a). In a conventional ISFET, the binding sites at the solid-liquid interface adsorb or release H^+^ ions, but selective sensitivity to different ions can be achieved as well by suitable surface functionalizations [6,7,8]. As described by the site binding (SB) model [9], the binding of the H^+^ ions induces a surface charge that is balanced by a spatial rearrangement of the ions in the electrolyte. This causes a potential drop at the sensing interface that is transduced into a threshold voltage (V_TH_) shift of the underlying FET. The pH-to-bias conversion factor follows the Nernst law, with a maximum ΔV_TH_ at room temperature of about 60 mV per pH point variation in the LUT, provided the sensing surface can perfectly buffer the pH of the solution at the interface (pH_s_). 

Unfortunately, as device dimensions are downscaled, the electrical and chemical noise is more likely to impact the final reading, thus affecting the sensor’s resolution [10]. To improve the resolution and achieve reproducible measurements, a detailed understanding of the DC and transient device behavior is necessary; insight on the dominant noise sources should be gained as well.

In this regard, it has often been reported that the electrical noise of the FET underlying any ISFET is much larger than the so-called chemical noise (i.e., the noise related to the stochastic binding/unbinding of the target ions), with the latter source of noise then being neglected [11,12,13,14,15,16]. However, recently published models [17,18,19,20] show that when the chemical noise can be observed on top of the FET intrinsic noise, then precious information on the biological transduction can be extracted from measurements. Furthermore, few works also claimed successful experimental evidence of chemical noise [19,21,22].

In this paper, we focus on the characterization and modeling of an ISFET pH sensor based on an industrial CMOS node, with particular interest in the origin and importance of the low-frequency noise. Firstly, we provide a methodology to calibrate the model of the ISFET in static, transient, and AC conditions, based on the device’s electrical characteristics and on the SB reactions taking place at the sensing layer. Then, we emphasize the qualitative and quantitative role played by the chemical noise in our experiments. The device vehicle for our study is the foundry-made FET with the in-house BEOL modification presented in Reference [23]; the BEOL modification uses a single photolithographic mask to remove the oxide and nitride passivation layer on the vertically-extended sensing gates through reactive ion etching (RIE) and subsequently performs lift-off of a platinum layer on the same areas. Vertical extension of the metal gates is included in the foundry process and drastically simplifies the in-house post-processing, reducing the depth of the trenches needed and removing the necessity of landing on a thin layer of the gate dielectric. This kind of device represents a promising candidate for industrial production of ISFET-based wearable sensors: Previous devices, in fact, were either entirely fabricated in-house in research labs with a long, expensive, and low-yield process [24,25,26], or purely industrially produced (foundry process), using the default nitride passivation as sensing layer, resulting in low sensitivity, high V_TH_ spread and remarkable drift [27,28,29].

## 2. Materials and Methods

In the following, the baseline device used for the characterizations, the experimental methods employed for the extraction of the electrical and electro-chemical parameters, and the models developed to explain the results are outlined individually.

### 2.1. Foundry-Made, BEOL-Modified ISFET

The investigated baseline FET is industrially fabricated with a standard CMOS process in the 0.18 µm technology node. The device features a 4 nm thick SiO_2_ gate dielectric on top of bulk silicon. The polysilicon gate contact has a surface of 10 µm times 20 µm and is extended through the SiO_2_ passivation layers with a series of vias (Figure 1b) to a top metal layer made of aluminum and featuring the same dimensions. The fabricated sensing chip (Figure 1c) features 45 identical ISFETs divided into five groups of nine, plus a metal contact meant for the definition of a miniaturized RE (mRE).

The Si_3_N_4_ passivation layer on top of the Al metal layer deposited by the foundry process is then locally removed in the BEOL through standard photolithography, and RIE carried out in our lab. The same photolithographic mask is then used to lift-off a 100 nm thick layer of platinum, to suppress unwanted chemical reactions at the sensing interface. Using the original Al layer as a sensing surface, in fact, could also be a suitable option for the definition of a sensor, especially thanks to the appearance of a native Al_2_O_3_ layer, which would provide for a near-Nernstian sensitivity. However, Al is a chemically active material that could dissolve in the LUT, generating undesirable and poorly controlled secondary effects. A possible and more sensitive alternative to platinum could have been the atomic layer deposition (ALD) of a high-k dielectric, but the deposition of an insulating layer on the chip would require an additional photolithographic mask in the BEOL to recover the electric contacts. Moreover, possible adverse effects, such as the appearance of trapped charges, due to the poor interface for growth of the dielectric, have not yet been investigated for this device, and are out of the scope of this work.

An mRE has been defined on an ad-hoc made contact on top of the sensing chip by lift-off of Pt followed by manual deposition of a silver paste. The metal stack is then covered with a protective polyvinyl butyral (PVB) membrane with an approximative thickness of 3 to 5 µm [30]. The resulting mRE, tested against a commercial RE (MicruX Ag/AgCl Reference Electrode) with an open circuit potential (OCP) measurement, showed a drift as low as 73 mV over 5000 s, about 75% of which is resolved after the first 5 min.

### 2.2. Experimental Methods

**Figure 2:**
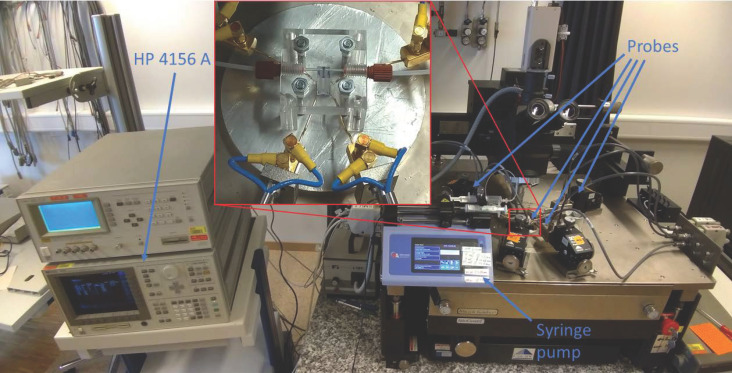
Measurement setup for the characterization of the BEOL ISFET. The HP 4156 A (on the left) applies the bias inputs and reads the current outputs from the device under test (DUT) through the four probes (on the right). A syringe pump provides a continuous flow of liquid on the chip through the PMMA microfluidics mounted on the chip (inset).

All measurements featuring an LG have been carried out using custom-made PMMA microfluidics sealed on top of the sensing chip, to prevent evaporation and ensure a constant flowrate when needed. The pH buffers are flown in the microfluidics using an 11 Elite Harvard apparatus syringe pump. All electrical measurements have been performed employing an HP 4156 A Precision Semiconductor Parameter Analyzer which, as discussed in Reference [31], is a suitable option not only for DC characterization, but also for sampling the current waveforms needed to compute the low-frequency noise spectra. The complete setup is shown in Figure 2.

Three types of measurements were made: (i) Double sweeps of the mRE bias to extract the transfer characteristics of the ISFETs, (ii) continuous sampling of the drain current, while switching from one pH buffer to another, and (iii) continuous sampling of the drain and gate (mRE for ISFET, metal contact for FET configuration) currents at different sampling rates to investigate the noise. In all cases, the bulk of the chip has been connected to the ground, and a fixed drain to source voltage of 100 mV has been used.

**Figure 3:**
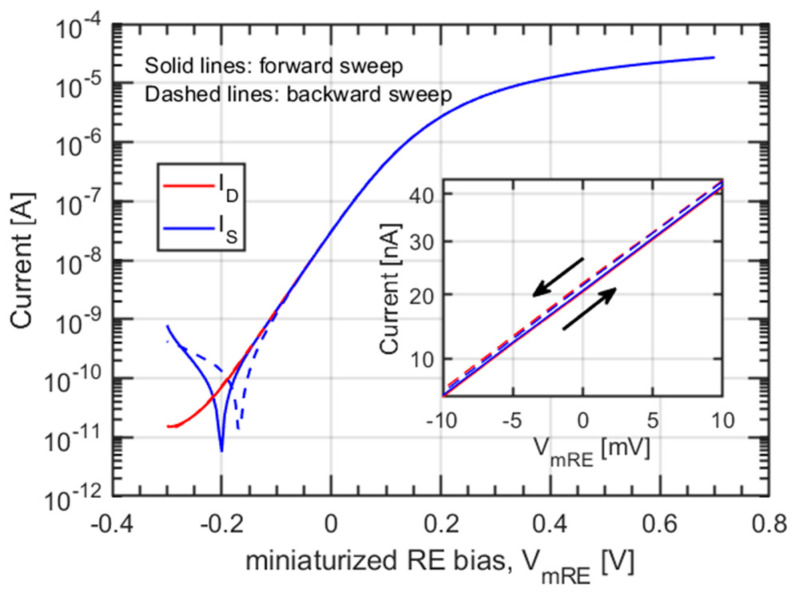
Double-sweep transfer characteristics, I_D_-V_mRE_, of the ISFET under characterization for an electrolyte with pH = 7. The inset on the right shows the ultralow (<1 mV) measured hysteresis.

The measured transfer characteristics (Figure 3) were used to investigate the level of hysteresis of our sensors, the gate bias levels needed to set them in a specific condition (subthreshold or strong inversion), and their transconductance, which is necessary to interpret the noise measurements at different biases. The transfer characteristics have also been measured with different pH buffers to investigate the sensitivity of the Pt sensing layer. Between two consecutive measurements, the channels have been emptied using an empty syringe, cleaned with DI water flown with the syringe pump, and emptied again. The transfer characteristics are extracted after 5 min of continuous flow at a rate of 10 µl/min of the pH buffer. The pH 4 buffer solution was prepared by mixing 1 M solution of sodium citrate dihydrate with a 1 M solution of citric acid (1:2.6 proportion), and then diluting the resulting buffer to 50 mm. Analogously, the pH 6, 7, and 8 buffers were prepared by mixing 1 M solution of disodium hydrogen phosphate with 1 M solution of sodium dihydrogen phosphate (1:7.3 proportion for pH 6, 1.36:1 proportion for pH 7, and 13.7:1 proportion for pH 8), and then diluting the resulting buffers down to 50 mm. From the electrostatic and surface chemistry point of view addressed in this work, moving to different buffers affects the sensor’s response only if the ionic strength also changes. Further details on the importance of this parameter are given in Section 2.3.3.

**Figure 4:**
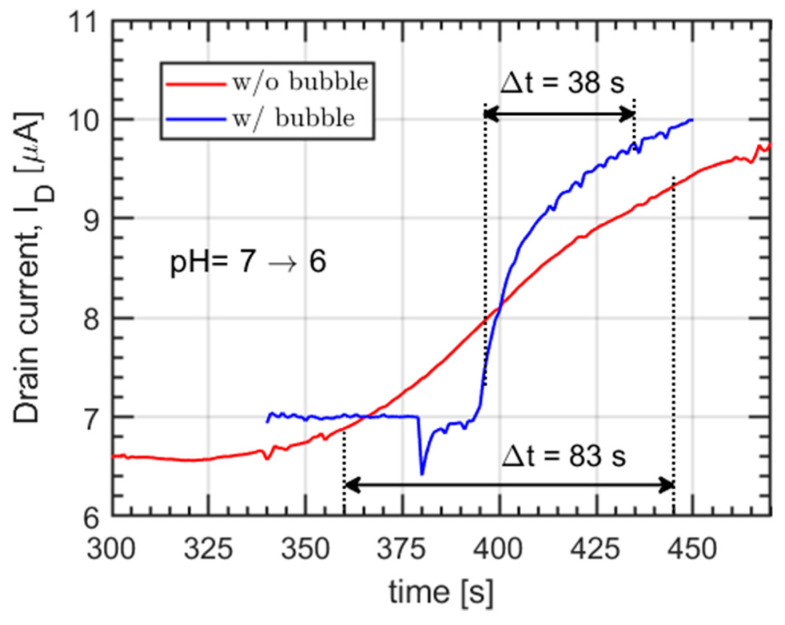
Comparison between the drain current, I_D_, response over time with and without the employed air bubble strategy to prevent interdiffusion. The 10% to 90% transition times are indicated in the plot with dashed lines, for the two cases, respectively.

Data from a continuous sampling of the drain current, while changing pH buffer has been used to calibrate the model of the site-binding kinetics at the sensing layer, i.e., the metal surface in contact with the electrolyte. A low-pressure stream selector (Cheminert C25-3188) has been used together with the syringe pump to switch from one pH buffer to another without interrupting the flow. This kind of measurement is challenging, since it is necessary to prevent two subsequent liquids from mixing in the channel by diffusion, adding an unknown contribution to the response time. In this work, the problem has been solved by briefly switching the selector to an empty channel, between two consecutive pH buffers, allowing a very thin air bubble (equivalent volume <0.5 µL, according to the set flow rate and switching time) to form, preventing mixing. The effectiveness of this technique is demonstrated by the readings, shown in Figure 4, in which the transient response to a pH change from 7 to 6 is shown on the same device with and without the creation of a small bubble in between.

It appears that, in the latter case, the interdiffusion affects the reading generating a smooth, slow transition from one value to another, while the presence of the bubble determines a steep and abrupt variation of the drain current.

The long-term sampling of the drain and gate currents with a fixed pH buffer and no flow has been performed to extract their respective noise power spectral densities (PSDs) in both FET and ISFET configuration. The comparison between the extracted PSDs allowed us to investigate the main sources of noise in the two architectures. The parameter analyzer employed could record a maximum of 5000 sample measurements; therefore, to expand the spectrum over which we can investigate the PSD, we performed for each configuration two consecutive measurements: One with a long sampling interval (1 s) and another with a short one (42 ms unless otherwise stated). It should be noted that for some measurements, the shorter sampling interval resulted in being insufficient for the integration time needed by the instrument, in which case it was automatically doubled to 84 ms.

**Figure 5:**
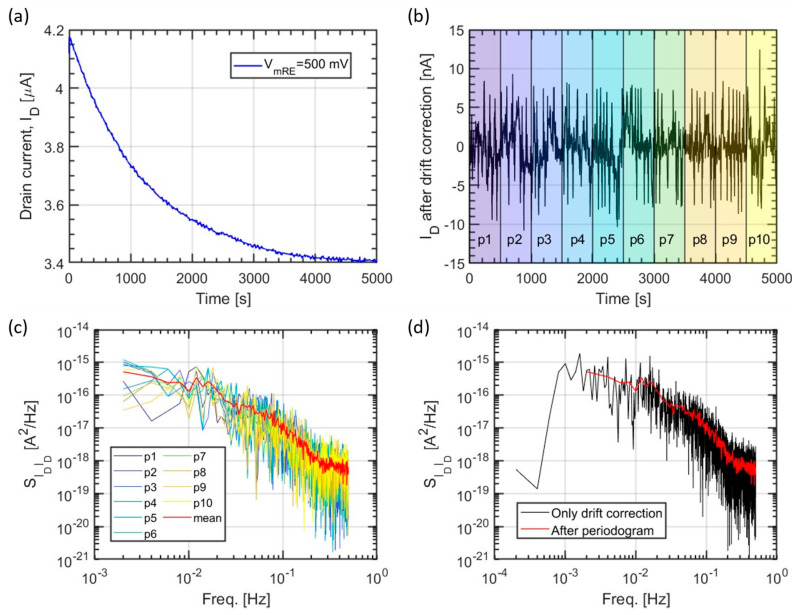
Example of post-processing a drain current waveform measured with sampling time $T_{S}=1$ s and $V_{mRE}=500$ mV. (**a**) The measured raw waveform of the drain current vs. time. (**b**) The same waveform after drift removal using a 10th order polynomial function and subdivision in ten portions (colored areas) for the application of the periodogram technique. (**c**) Power spectral density (PSD) of each portion of the drift-corrected drain current waveform in (**b**) with a red line showing the average between all the PSDs, i.e., the periodogram. (**d**) Comparison of the PSDs with data cleaned with only the drift correction and data cleaned with both drift correction and periodogram.

The observed slow drift of the sensor current (probably due to absorption of H^+^ ions in the bulk of the Pt layer, as well as deposition of electrolyte ions on the Pt surface) needs to be considered when evaluating the noise PSDs, especially for long-term measurements, or it will drastically influence the results of the fast Fourier transform (FFT) calculations. Figure 5a shows the typical raw data of the ISFET drain current sampling obtained in strong inversion. The drift is clearly higher than the noise of the reading. To remove it, we have subtracted the 10th order polynomial fit of the curve to the raw trace (Figure 5b). The curve can be smoothened further, at the cost of a reduced frequency range and resolution, using the periodogram technique. For example, we divide the reading into ten equal portions, and extract the average of their respective PSDs (Figure 5c). The PSDs after drift correction alone and drift correction plus periodogram are compared in Figure 5d.

### 2.3. Models and Simulation Methodology

#### 2.3.1. Analysis of Static (DC) Measurements

**Figure 6:**
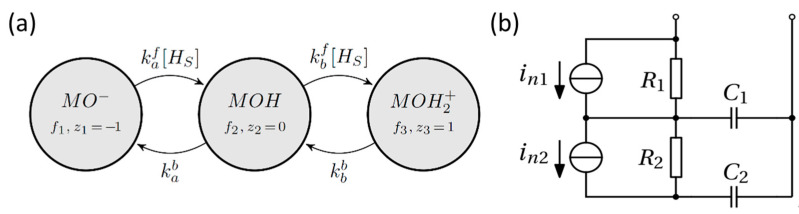
(**a**) Kinetic diagram of the site binding (SB) model from Reference [20]. The graph shows two chained reactions involving the protonation-deprotonation of one hydroxyl binding site. Each state is characterized by a net signed number of elementary charges, z, and a probability of the site to be in that state, f. The coefficients on the transitions arrows relate the probabilities of the two adjacent states. ($k_{a}^{f}$; $k_{a}^{b}$) and ($k_{b}^{f}$; $k_{b}^{b}$) are the forward and backward reaction constants for the first and second protonation reaction, respectively. (**b**) Equivalent noise circuit of the SB reactions in (**a**). The chemical noise is represented by the thermal noise of the resistors, here shown as Norton’s equivalent current generators.

Changes of the pH buffer cause shifts of V_TH_, due to surface charge developed at the sensing layer surface. The pH-induced V_TH_ modulation in ISFETs is usually described using the first order SB model, whose kinetic form is illustrated in Figure 6 [20]. The model considers at least two chained surface reactions involving the hydrogen ions at the surface (with concentration $\left[H_{S}\right]$) and the exposed surface hydroxyl groups in the complexation states MO^−^, MOH, and MOH_2_^+^. At equilibrium, the surface charge and the shift of the FET threshold are defined by the pH in the bulk of the solution, the density of surface sites $N_{S}$, and the reactions’ dissociation constants, here defined as the ratios between backward and forward reaction rates: $K_{a}=k_{a}^{b}/k_{a}^{f}$ and $K_{b}=k_{b}^{b}/k_{b}^{f}$. Hence, the surface charge density developed at the sensing layer can be expressed, as shown in Reference [9],

(1) $$Q_{S}=qN_{S}\frac{{\left[H_{S}\right]}^{2}-K_{a}K_{b}}{{\left[H_{S}\right]}^{2}+K_{b}\left[H_{S}\right]+K_{a}K_{b}},$$

where q is the elementary charge.

Given the electrolyte composition, we used the data of the V_TH_ shifts to calibrate the $K_{a}$, $K_{b}$ and $N_{S}$ parameters of the SB model, using a model implementation that solves self-consistently the electrostatics of the sensing surface and of the electrolyte system. The details of the equations used can be found in Reference [32].

#### 2.3.2. Transient Analysis

Calibration of the SB model under equilibrium conditions only provides the dissociation constants $K_{a}$ and $K_{b}$ which do not describe the dynamic over time of the binding/unbinding process. The complete extraction of the kinetic SB model parameters can only be obtained by fitting a transient response [33,34]. The model in Figure 6a is, thus, calibrated by comparison with the current waveforms extracted when switching the pH. From Reference [20], the master equation of the SB model, shown in Figure 6a, reads

(2) $$\{\begin{array}{c} \frac{df_{1}\left(t\right)}{dt}=-k_{a}^{f}\left[H_{S}\right]f_{1}\left(t\right)+k_{a}^{b}f_{2}\left(t\right)\\ \frac{df_{2}\left(t\right)}{dt}=k_{a}^{f}\left[H_{S}\right]f_{1}\left(t\right)-\left(k_{a}^{b}+k_{b}^{f}\left[H_{S}\right]\right)f_{2}\left(t\right)+k_{b}^{b}f_{3}\left(t\right)\\ \frac{df_{3}\left(t\right)}{dt}=k_{b}^{f}\left[H_{S}\right]f_{2}\left(t\right)-k_{b}^{b}f_{3}\left(t\right) \end{array},$$

whereas the net surface charge density for $N_{S}$ identical sites per unit of surface area is:

(3) $$Q_{S}\left(t\right)=qN_{S}\left(z_{3}f_{3}\left(t\right)+z_{1}f_{1}\left(t\right)\right).$$

Since the sum of the occupation probabilities must be equal to 1, one can simplify Equation (2) substituting $f_{2}$ in terms of $f_{1}$ and $f_{3}$. The resulting two-equations system reads:

(4) $$\frac{df^{\prime }\left(t\right)}{dt}=A\cdot f^{\prime }\left(t\right)+b,$$

where $f^{\prime }\left(t\right)$ is a column vector containing $f_{1}\left(t\right)$ and $f_{3}\left(t\right)$ as elements, A is a 2 × 2 matrix containing the coefficients that multiply with the occupation functions, and b is a 2-elements column vector of the remaining parameters.

We used the backward Euler method to discretize and solve Equation (4), obtaining a linear relationship between future and previous estimates of the state probability,

(5) $$f^{\prime }\left(t+\Delta t\right)={\left(I_{2}-\Delta tA\right)}^{-1}\cdot \left(f^{\prime }\left(t\right)+\Delta tb\right),$$

where Δt is the time step and $I_{2}$ is the 2 × 2 identity matrix, which results after the discretization of the time derivative and subsequent factoring. Equation (3) then yields the transient variations of the surface charge. It is important to note that the terms A and b should be updated at each time step, due to the electrostatic coupling between surface charge and ionic species concentrations at the surface (e.g., $\left[H_{S}\right]$). This task is significantly simplified when the double layer capacitance, $C_{DL}$, does not change appreciably between two pH buffers, a condition that has been numerically verified for the cases considered in this work. Hence, by using Boltzmann statistics for the description of ion distributions in the electrolyte (no steric effects), the surface potential at the sensing layer, $V_{S}$, can be calculated as

(6) $$V_{S}\approx \frac{Q_{S}}{C_{DL}}.$$

We then plug $V_{S}$ into the Boltzmann equation to calculate a new estimate of $\left[H_{S}\right]$,

(7) $$\left[H_{S}\right]=\left[H_{B}\right]e^{\left(-V_{S}/V_{th}\right)},$$

where $\left[H_{B}\right]$ is the bulk hydrogen ions concentration and $V_{th}=k_{B}T/q$. The backward and forward reaction constants of the acid and basic reactions are used as fitting parameters to reproduce the experimental current transients corresponding to pH steps. Each of them represents a time constant τ according to $\tau_{a}=1/k_{a}^{b}$ and $\tau_{b}=1/k_{b}^{b}$. The forward reaction constants are then given from the definition of equilibrium dissociation constants calibrated from the static measurements, which is $k_{a}^{f}=1/\left(K_{a}\tau_{a}\right)$ and $k_{b}^{f}=1/\left(K_{b}\tau_{b}\right)$, respectively.

#### 2.3.3. Noise Spectra Analysis

**Figure 7:**
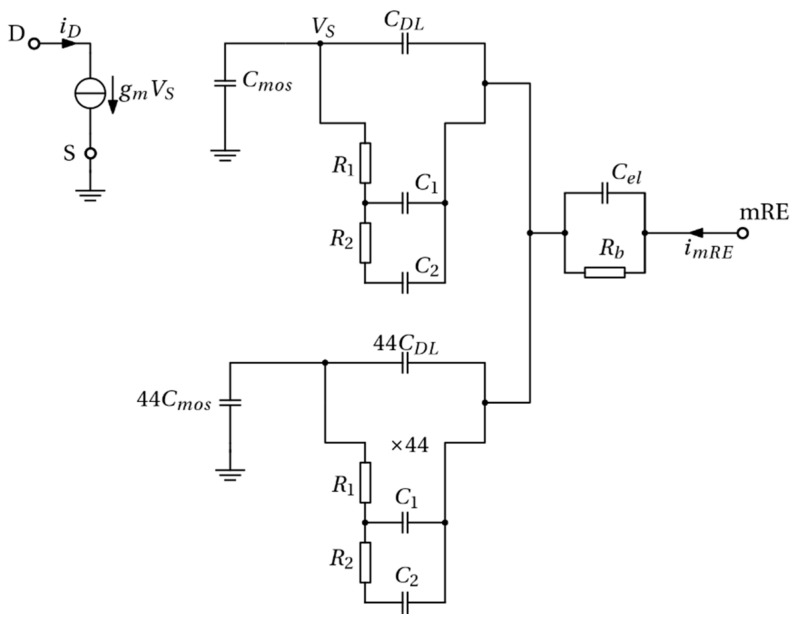
Complete circuit model of one active ISFET (top left portion) and 44 unbiased ones (source and drain floating, but substrate grounded) in parallel (bottom left). The equivalent circuit model of the surface impedance, due to the SB reactions is given by a two-cell network consisting of $R_{1}$, $R_{2}$, $C_{1}$ and $C_{2}$ lumped elements (see Figure 6b). The bulk of the electrolyte is represented by the parallel between $C_{el}$ and $R_{b}$, whereas the mRE is assumed ideal. The node $V_{S}$ indicates the sensing layer of the active ISFET. The simulation includes the thermal noise of resistors of $R_{b}$ and of $R_{1}$ and $R_{2}$ as indicated in Figure 6b.

Fluctuations of the surface charge at the sensing layer can be calculated from the master equation, as presented in Reference [20]. In particular, it is possible to directly link the master equation to the surface charge density noise PSD or to an equivalent circuit of the surface reactions. The latter method was chosen, since it can be included in the circuit representation of the entire electro-chemical system. The equivalent circuit of the SB-like model consists of a two-cells ladder network (see Figure 6b), where each cell is the series of a resistor and a capacitor [19,20], and the chemical noise at the single port is given by the thermal noise of the resistive components, i.e., $R_{1}$ and $R_{2}$. This network must be placed parallel to the double layer capacitance in the complete model, as shown in Figure 7. Following [20], we obtain the following symbolic expressions of the lumped elements $R_{1}$, $R_{2}$, $C_{1}$ and $C_{2}$:

(8) $$R_{1}=\frac{V_{th}\left({\left[H_{S}\right]}^{2}+K_{b}\left[H_{S}\right]+K_{a}K_{b}\right)}{qWLN_{S}\alpha }\;C_{1}=\frac{qWLN_{S}\alpha^{2}}{\left[H_{s}\right]K_{b}\beta }\;R_{2}=\frac{\beta }{qWLN_{S}\left[H_{s}\right]K_{a}k_{a}^{f}k_{b}^{f}\alpha \gamma }\;C_{2}=\frac{qWLN_{S}{\left[H_{s}\right]}^{2}K_{a}K_{b}\gamma }{\beta }\;\mathrm{with}\;\alpha =\left[H_{s}\right]K_{b}\left(\left[H_{S}\right]k_{b}^{f}+K_{a}k_{a}^{f}\right)\;\beta =V_{th}\left({\left[H_{s}\right]}^{2}+K_{b}\left[H_{s}\right]+K_{a}K_{b}\right)\cdot {\left({\left(\left[H_{S}\right]k_{b}^{f}\right)}^{2}+\left[H_{s}\right]K_{a}{\left(k_{a}^{f}\right)}^{2}-2\left[H_{s}\right]K_{a}k_{a}^{f}k_{b}^{f}+K_{b}\left[H_{s}\right]{\left(k_{b}^{f}\right)}^{2}+{\left(K_{a}k_{a}^{f}\right)}^{2}\right)}^{2}.\;\;\gamma ={\left(\left[H_{s}\right]k_{a}^{f}-2\left[H_{s}\right]k_{b}^{f}+2K_{a}k_{a}^{f}-K_{b}k_{b}^{f}\right)}^{2}.$$

The BEOL-modified chip is equipped by 45 ISFETs (Figure 1c) exposed to the electrolyte medium. Only one ISFET is operated at a time (i.e., source and drain are biased), but all of them have the substrate connected to the ground so that binding/unbinding of ions at these additional ISFETs may induce voltage fluctuations as well. As a result, the global equivalent circuit of a noiseless device with an ideal mRE is the one in Figure 7.

The drain current of the active ISFET is proportional to the potential at the sensing Pt layer, through the transconductance $g_{m}$. The bulk of the electrolyte is represented by the parallel connection of $C_{el}$ and $R_{b}$, whose values were estimated to be $C_{el}=1.88$ pF and $R_{b}=127$ Ω, based on the background ionic strength of the buffer and the system geometry. The noise generated by the resistor $R_{b}$ was also included in the model, but was found to have a negligible effect in the frequency range of interest. A double layer capacitance $C_{DL}=102.6$ pF was extracted from self-consistent PB calculations (model in Reference [32]). Finally, the FET capacitance was estimated to be $C_{mos}=0.9$ pF from CV measurements (not shown). This was in fair agreement with the calculations based on physical and geometrical factors of the gate stack. Furthermore, extensive simulations showed that the noise at the node at the sensing layer, $V_{S}$, (see Figure 7) in the frequency range of interests in this work was quite insensitive to changes in $C_{mos}$, $C_{el}$ and $R_{b}$ and to the presence of the 44 unbiased ISFETs. In fact, the noise generated by the surface reactions was essentially short circuited by the double layer capacitance $C_{DL}$, which mainly depends on the ionic strength. However, changing the ionic strength of the buffer composition has not just an impact on $C_{DL}$, but also on all the component values in Figure 7, except from $C_{mos}$. While this is trivial for the bulk electrolyte resistance, $R_{b}$, and capacitance, $C_{el}$, it may not be straightforward from the equivalent noise circuit components of the SB reactions, as shown in Equation (8). Here, the role of the background ionic concentration of the electrolyte is on the electrostatics, which determines the concentration of hydrogen ions at the sensing layer, $\left[H_{S}\right]$ [see Equation (7)].

## 3. Results and Discussion

In this section, we report the results of the different sets of measurements introduced previously, describing in detail the data processing (if any) and analysis used to generate the figures. Modeling results are also shown for each part of the characterization.

### 3.1. Transfer Characteristics

The transfer characteristic of the fabricated ISFET is shown in Figure 3. The I_ON_/I_OFF_ ratio exceeds 10^6^, with a nearly ideal subthreshold swing (SS) of 75 mV/dec. The double-sweep measurement highlights a modest difference between the transfer characteristics in a forward and backward sweep. A hysteretic device performs poorly as a sensor, since its readings are influenced by the previous measurements, which results in poor repeatability. This effect is usually due to defects (active electrical traps) in the dielectric gate stack and/or at the interfaces of the oxide with the semiconductor [26]. The inset of Figure 3 shows that our device, thanks to the industry-level fabrication process, presents a negligible hysteresis of less than 1 mV, and is, therefore, suitable for sensing.

**Figure 8:**
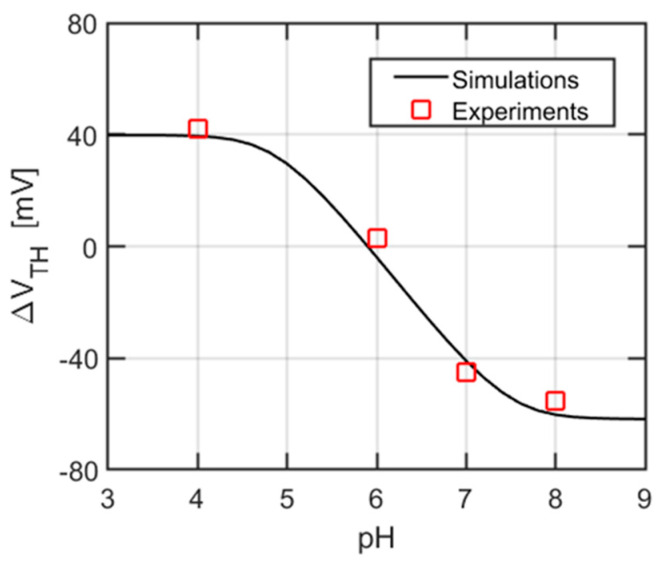
Threshold voltage variation with pH and simulation results obtained fitting the associated SB model.

The variation of the threshold voltage (V_TH_), due to a change of pH buffer is reported in Figure 8, with a maximum slope of ca. 40 mV/pH around pH = 6, namely, the point of maximum sensitivity. In line with previous findings [35,36,37,38], Pt sensing layers have a smaller pH sensitivity compared to high-*k* dielectrics, such as Hafnia and Alumina (57 mV/pH for HfO_2_ [26] and 59 mV/pH for Al_2_O_3_ [6]), and also spans a reduced pH range. We also note that among ISFETs with Pt sensing layers, significant differences are expected according to the degree of Pt surface oxidation [6,35], with oxidized samples showing a higher surface density of binding sites.

Figure 8 also reports the simulations using the DC model described in Section 2.3.1. The set of parameters yielding the best fit with the experimental data are reported in Table 1. To the best of our knowledge, this is the first time that the SB model is calibrated to fit the pH response of a platinum layer.

### 3.2. Transient Response

**Figure 9:**
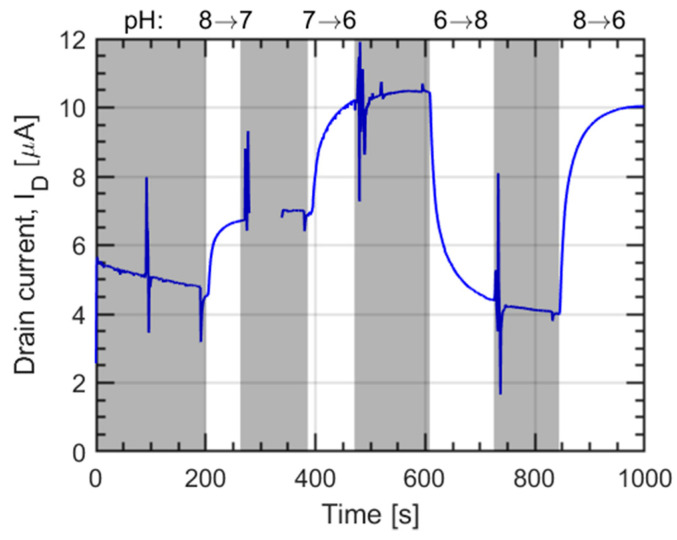
Drain current response vs. time of a set of pH transitions. The areas not relevant for the characterization of the transition have been greyed out.

Three different buffers at pH = 6, 7, and 8 have been used to characterize the transient response of the sensing layer. The transition of one and two pH points in both ascending and descending order have been characterized. The results are shown in Figure 9.

We observe that, especially for pH = 8, the readings are affected by drift, which forced us to select a reasonable threshold after which the transition is assumed completed. The chosen flag to assess this was the convergence of the measurement to a rate of change comparable with the drift observed in the first 200 s of measurement. After the first transition from pH 8 to 7, the probe contact with the drain has been shortly lost, which is why an empty section in the second greyed out a portion of our graph is observed. A set of spikes can also be observed at a constant time interval before the start of the next transition: This is due to the external manipulation of the stream selector to switch the buffer which is flown on the sensor. The delay before the change of drain current is due to the time needed by the new buffer to reach the sensing area through the microfluidics, as demonstrated by the short spike observed right before the transition, which signals the passage of the small bubble. Furthermore, the entity of the delay (around 20 s) is in line with the time needed to fill a 10 cm long tube with a diameter of 0.5 mm using a flow rate of 10 µl/min.

**Figure 10:**
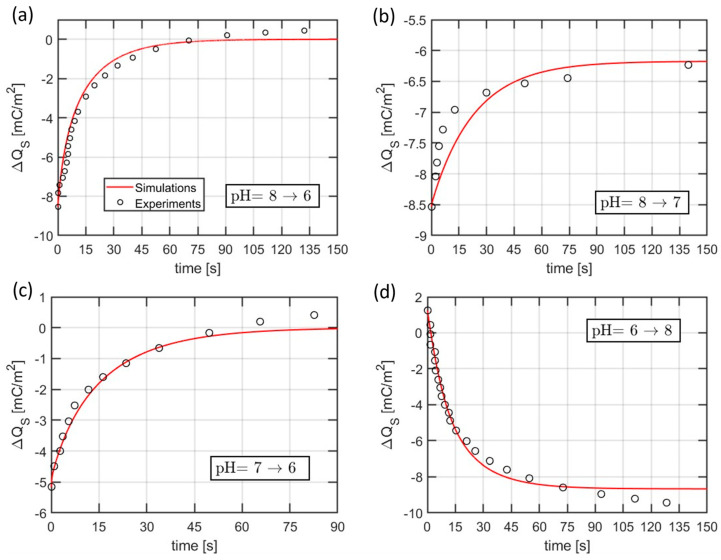
Model calibration from experimental data in terms of transient surface charge changes upon pH step. The experimental data are taken from the current transients in Figure 9 and are scaled using an arbitrary linear relationship between surface charge and drain current. The values of the employed time constants $\tau_{a}$ and $\tau_{b}$ are, respectively, 9.5 and 7 s.

By assuming that the drain current is directly proportional to the surface charge at the interface between the sensing layer and the electrolyte (see Section 2.3.2), the transient curves in Figure 9 can be directly interpreted in terms of surface charge variations as predicted by the SB model. Since the goal is to determine the time constants, the current has been converted to the surface charge density $Q_{s}$ via a scaling factor, such as to span the same range as in the simulations. Figure 10 reports the comparison between experiments in Figure 9 and simulations employing the set of time constants $\tau_{a}$ and $\tau_{b}$ yielding the best fit that reproduces the initial portion of all the transients, which is expected to be less affected by spurious drifts.

### 3.3. Noise Characterization

**Figure 11:**
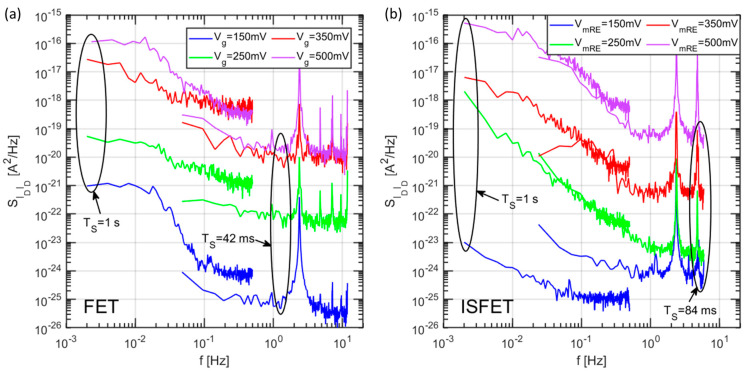
Drain current PSDs extracted at different gate biases for the FET (**a**) and ISFET (**b**), configuration of the same device. Different sampling rates are shown for each bias. The buffer used for the liquid gate of the ISFET configuration has pH = 7.

The noise PSDs for the drain current of the same device in the FET and ISFET configuration have been extracted at a few gate biases ranging from near threshold to inversion conditions. The results, post-processed with the method described in Section 2.2, are shown in Figure 11.

As can be seen from our experiments, a first important observation is that a very comparable level of drain current noise is observed in both FET and ISFET configuration (measurements with liquid gate) once the drift predominantly affecting the liquid gate device, is removed. A strong influence of the gate bias is observed, due to the higher level of currents for higher biases. The measurements at higher sampling rates displayed several peaks in the frequency range between 1–10 Hz, which we could unambiguously attribute to aliasing at the low frequency of the 50 Hz component from the power supply. A difference is sometimes observed between the PSDs at equal bias for long and short sampling intervals, in the common frequency range, which could also be attributed to aliasing of the noise at frequencies higher than the Nyquist frequency.

**Figure 12:**
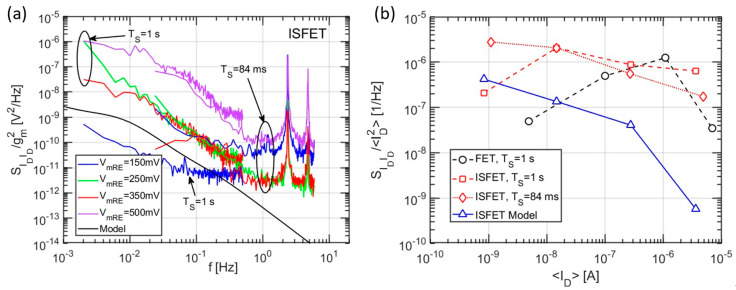
(**a**) comparison of the input voltage referred noise PSD at the sensing layer obtained from experiments and from the circuit simulation of Figure 7. (**b**) Normalized PSD at 0.1 Hz for FET and ISFET configuration of the same device, together with the ISFET model results in (**a**).

To compare the model shown in Section 2.3.3 with the drain current noise of Figure 11, we convert the latter into a voltage noise at the sensing layer using the FET transconductance as transfer function: $S_{V_{g}V_{g}}=S_{I_{D}I_{D}}/g_{m}^{2}$. The result of this transformation is compared to the noise simulations in Figure 12a for different gate voltages. We see that the simulated noise PSD is in line with the experiments at low bias, showing a similar noise level and a knee approximately at the same position that is an indication of the dominant time constant of the binding/unbinding process at the sensing layer. On the other hand, at high biases, the measured drain noise PSD is way larger than the simulated one, which includes only the chemical noise related to stochastic binding/unbinding of H^+^ ions.

To better investigate this point, the PSDs at 0.1 Hz have been normalized with respect to the average steady-state current, as shown in Figure 12b. At low drain currents, the model is in good agreement with the experimental data of the normalized ISFET noise (red curves). However, the latter shows a flat-like profile, while scaling the drain current, in contrast with the model predictions where a down-scaling as $g_{m}^{2}/I_{D}^{2}$ is found. According to Reference [39], this suggests that the carrier number fluctuations (assumed by our model of chemical noise [20]) are dominant only at low currents, whereas in the high current regime, an additional mechanism may dominate the drain current noise. In particular, the similar noise values shown by the FET and ISFET further suggest that this additional noise source takes place in the underlying FET instead that in the electro-chemical domain of the ISFET.

**Figure 13:**
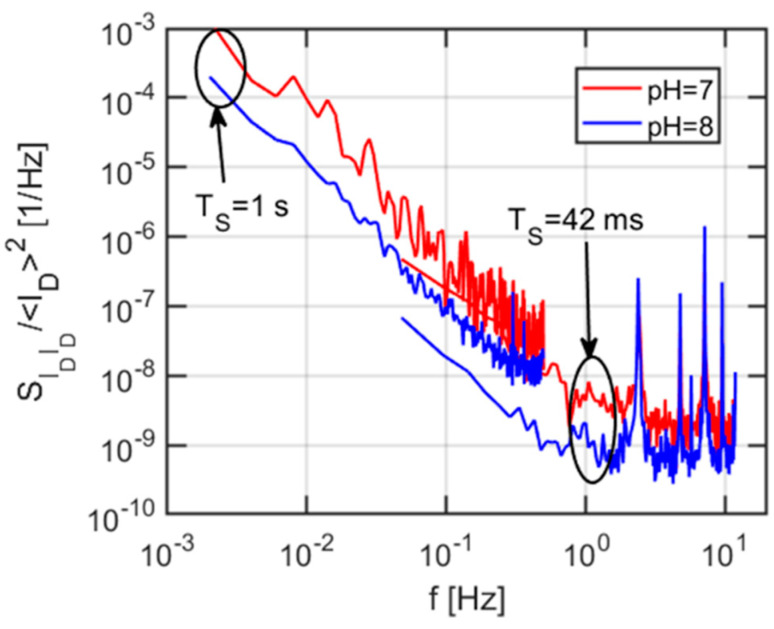
Normalized drain current noise PSD using two different pH buffers.

To test the influence of the liquid gate composition, a normalized PSD has also been extracted for two different pH buffers (pH 7 and pH 8). The results, shown in Figure 13, suggest that, while the PSDs are almost identical at higher frequencies, some differences are observed at frequencies lower than 1 Hz. As reported in Reference [36], such differences could be ascribed to changes in the oxidation state of the Pt layer, caused by undesired redox agents or interfering substances reacting at the surface.

**Figure 14:**
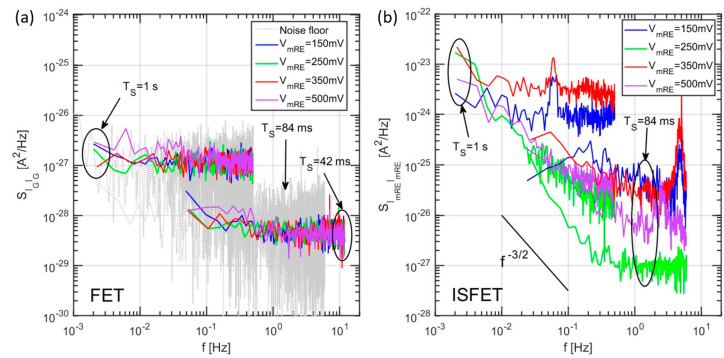
Noise PSDs of the metal (**a**) and liquid gate (**b**) current. The floor PSD is evaluated from an open-circuit measurement of the setup, performed applying to each probe the same bias which was used for the characterization of the leakage currents. Note that different y-axis scales are used in (**a**,**b**), respectively.

Noise PSD measurements have also been carried out on the leakage currents through the metal and liquid gates of the same device, with results shown in Figure 14.

Contrary to the previous measurements, here we notice a clear difference between the two graphs: In particular, the FET intrinsic noise appears to be below the limit of detection of our characterization system (shown in grey, without periodogram). We also observe that the values extracted at low and high sampling rate have a ratio identical to the one between the sampling times, pointing at a strong influence of aliasing on our readings, at this level of noise. On the other hand, the device in the ISFET configuration displays a large current noise at the mRE, which has a weak dependence on the bias, differently from the strong dependence of the drain current PSD on the mRE bias. This suggests a completely different mechanism. For example, in Reference [40], it was found that the noise current PSD, due to diffusion of ions should display a f^-3/2^ slope, which appears consistent with the data in Figure 14b.

**Figure 15:**
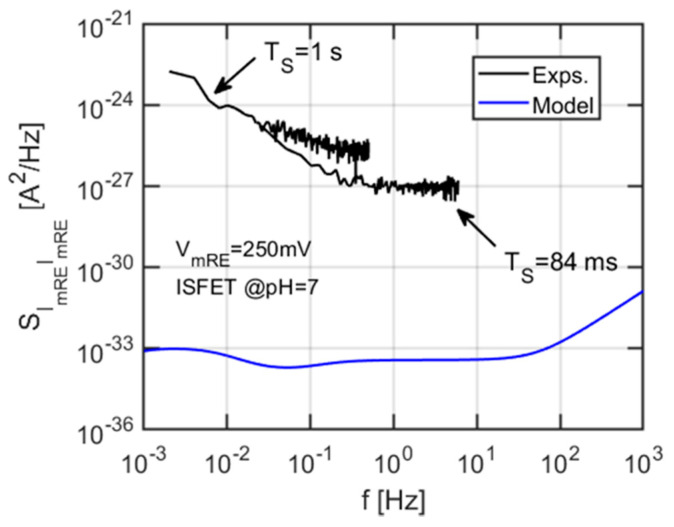
Comparison between the mRE current noise PSD from experiments (black lines, for two different sampling times) and the model with chemical noise at the sensing layer. It appears clear that the influence of the latter is negligible at the fluid gate.

For the sake of completeness, Figure 15 shows the comparison between the experimentally measured current noise PSD at the mRE and the simulations performed using the equivalent circuit, shown in Figure 7. The predicted chemical noise at the mRE is several decades lower than the measured one and has a completely different shape (with features associated with the various RC time constant in the equivalent circuit of Figure 7), since, in the model, the mRE current originates from capacitive coupling between the voltage fluctuations at the sensing layer (due to ions binding/unbinding) and the electrode. These marked differences between the experiments and the model for chemical noise suggest that other sources of noise, e.g., additional redox reactions at the reference electrode, random diffusion of ions, should be considered to explain the mRE current noise.

### 3.4. Device Resolution

To assess the device resolution, we start from the drain current noise PSD in Figure 11 and combine it to the voltage noise at the sensing layer (Figure 12a) and the pH response of the threshold voltage (Figure 8). In fact, the square root of the integral (over a reasonable frequency range, e.g., one decade) of the traces in Figure 12a gives the rms voltage fluctuations expected at the sensing layer,

(9) $$\sigma_{V_{S}}=\sqrt{\frac{\int^{\;}S_{I_{D}I_{D}}df}{g_{m}^{2}}}.$$

Considering a pH-sensitivity of 40 mV/pH (see Figure 8), we calculated the resolution of the device as $\sigma_{V_{S}}/40\mathrm{mV}$. Table 2 shows the results of this calculation for different mRE voltages and a normalization footprint area of 1 µm^2^ (i.e., the resolution is multiplied by $\sqrt{WL/1\;{\mu m}^{2}}=26.7$). We observe that, in the low current regime, corresponding to the ISFET subthreshold region of operation, the resolution is much better than in the high current regime. The best (smallest) resolution is in good agreement with that of similar devices in the literature [12,14,41,42,43,44,45]. Table 3 compares the resolution of this work with works based on other ISFETs in the literature, using the same evaluation criteria.

## 4. Conclusions

A careful, systematic analysis of pH sensitivity and noise measurements of BEOL modified CMOS foundry ISFETs with integrated reference microelectrode allowed us to gain insights on the sensitivity and resolution performance and limits of the considered technology. Although Pt is not the best possible material for pH sensing *per se*, good normalized pH resolution of 5.5 mpH μm2 with highly repeatable, nearly hysteresis-free characteristics was achieved with Pt sensing surfaces and the home-made reference microelectrode next to the device, close to the best results demonstrated in 481 published works employing high-k dielectrics as sensing layers. Experimental evidence was found that chemical binding/unbinding noise plays a role on the device resolution at very low current levels in the subthreshold region (weak inversion), but not when the device is biased above threshold (strong inversion). This kind of noise could possibly decrease with the adoption of a high-k dielectric as a sensing layer, due to the higher concentration of binding sites. No clear dependence of the measured noise on the pH of the solution was found in the investigated frequency range (2 MHz to 24 Hz). Indeed, simulations predict that a large double layer capacitance can significantly hinder the signature of the chemical noise in the low-frequency range. The analysis also led us to extract the site-binding model parameters of Pt. The home-made reference microelectrode does not appear to degrade the measured ISFET noise. However, a significant current noise was found at the mRE terminal of the ISFET that is orders of magnitude larger than the gate current noise of the underlying FET, and that is not explained by the chemical noise associated with the random binding/unbinding of hydrogen ions on the sensing layer. Understanding the origin of this additional noise would deserve future investigation, which goes beyond the scope of this work. Overall, this work has addressed noise considerations in the various regimes of operation of ISFETs, which paves the way for a better understanding and use in sensing applications of these devices.

## Figures and Tables

**Table 1:** Parameters values of the SB model for the Pt sensing layer, used to fit the experimental data shown in Figure 8.

Parameter.	Value	Units
$\mathrm{Dissociation}\;\mathrm{constant},\;K_{a}$	$10^{-5}$	mol/L
$\mathrm{Dissociation}\;\mathrm{constant},\;K_{b}$	$10^{-7}$	mol/L
$\mathrm{Density}\;\mathrm{of}\;\mathrm{sites},\;N_{S}$	$5.5\times 10^{16}$	m^−2^

**Table 2:** ISFET resolution to pH normalized to the standard footprint area of 1 ${\mu m}^{2}$, calculated for different fluid gate biases and average drain current values. The rms voltage noise at the sensing layer is calculated using Equation (9), where the drain current noise PSDs is taken from Figure 12a. For the case with $V_{mRE}$ = 150 mV, we used the trace sampled with $T_{S}$ = 84 ms and integrated over the frequency range 0.1–1.5 Hz. For all other cases, we used the traces sampled at $T_{S}$ = 1 s and integrated over the range 0.0301–0.4975 Hz.

Figure	Average Drain Current. <I_D_>	Normalized Resolution for $1\;\mu m^{2}$ (Units of pH)
150 mV	0.84 pA	0.0065
250 mV	14.7 nA	0.0051
350 mV	277 nA	0.0050
500 mV	4.85 µA	0.049

**Table 3:** Comparison of the resolution obtained in this work with other pH sensitive ISFETs in the literature.

Sensing Layer	Resolution (Units of pH)	Resolution for $1\;\mu m^{2}$ (Units of pH)	Bandwidth (Hz)	Center Frequency (Hz)	Ref.
SiO_2_	0.0008	0.0008	1	1	[14]
Al_2_O_3_	0.0005	0.0013	1	10	[44]
Al_2_O_3_	0.00017	0.001	1	10	[12]
Si_3_N_4_	0.010	0.044	-	-	[42]
Al_2_O_3_	0.0001	0.3	60	60	[43]
Si_3_N_4_	0.019	21.8	7.99	4	[45]
Pt	0.0002	0.0055	0.467	0.2635	This work

## Data Availability

The data presented in this study are available on request from the corresponding author.
